# Metabolic flux sampling predicts strain-dependent differences related to aroma production among commercial wine yeasts

**DOI:** 10.1186/s12934-021-01694-0

**Published:** 2021-10-21

**Authors:** William T. Scott, Eddy J. Smid, David E. Block, Richard A. Notebaart

**Affiliations:** 1 Department of Chemical Engineering, University of California, Davis, CA USA; 2 Food Microbiology, Wageningen University & Research, Wageningen, The Netherlands; 3 Department of Viticulture and Enology, University of California, Davis, CA USA

**Keywords:** Flux sampling, Genome-scale metabolic models, *Saccharomyces cerevisiae*, Volatile organic compounds, Wine

## Abstract

**Background:**

Metabolomics coupled with genome-scale metabolic modeling approaches have been employed recently to quantitatively analyze the physiological states of various organisms, including *Saccharomyces cerevisiae.* Although yeast physiology in laboratory strains is well-studied, the metabolic states under industrially relevant scenarios such as winemaking are still not sufficiently understood, especially as there is considerable variation in metabolism between commercial strains. To study the potential causes of strain-dependent variation in the production of volatile compounds during enological conditions, random flux sampling and statistical methods were used, along with experimental extracellular metabolite flux data to characterize the differences in predicted intracellular metabolic states between strains.

**Results:**

It was observed that four selected commercial wine yeast strains (Elixir, Opale, R2, and Uvaferm) produced variable amounts of key volatile organic compounds (VOCs). Principal component analysis was performed on extracellular metabolite data from the strains at three time points of cell cultivation (24, 58, and 144 h). Separation of the strains was observed at all three time points. Furthermore, Uvaferm at 24 h, for instance, was most associated with propanol and ethyl hexanoate. R2 was found to be associated with ethyl acetate and Opale could be associated with isobutanol while Elixir was most associated with phenylethanol and phenylethyl acetate. Constraint-based modeling (CBM) was employed using the latest genome-scale metabolic model of yeast (Yeast8) and random flux sampling was performed with experimentally derived fluxes at various stages of growth as constraints for the model. The flux sampling simulations allowed us to characterize intracellular metabolic flux states and illustrate the key parts of metabolism that likely determine the observed strain differences. Flux sampling determined that Uvaferm and Elixir are similar while R2 and Opale exhibited the highest degree of differences in the Ehrlich pathway and carbon metabolism, thereby causing strain-specific variation in VOC production. The model predictions also established the top 20 fluxes that relate to phenotypic strain variation (e.g. at 24 h). These fluxes indicated that Opale had a higher median flux for pyruvate decarboxylase reactions compared with the other strains. Conversely, R2 which was lower in all VOCs, had higher median fluxes going toward central metabolism. For Elixir and Uvaferm, the differences in metabolism were most evident in fluxes pertaining to transaminase and hexokinase associated reactions. The applied analysis of metabolic divergence unveiled strain-specific differences in yeast metabolism linked to fusel alcohol and ester production.

**Conclusions:**

Overall, this approach proved useful in elucidating key reactions in amino acid, carbon, and glycerophospholipid metabolism which suggest genetic divergence in activity in metabolic subsystems among these wine strains related to the observed differences in VOC formation. The findings in this study could steer more focused research endeavors in developing or selecting optimal aroma-producing yeast stains for winemaking and other types of alcoholic fermentations.

**Supplementary Information:**

The online version contains supplementary material available at 10.1186/s12934-021-01694-0.

## Introduction

The ability to produce wines with specific sensory profiles would be immensely beneficial to the global wine industry. In addition to characteristics coming from the grape juice, this optimal production is heavily contingent upon commercial yeast (*Saccharomyces cerevisiae* (*S. cerevisiae*)) strains to complete alcoholic fermentation and produce desirable aroma compounds, so-called volatile organic compounds (VOCs). It has been assessed that wines contain more than 1000 different VOCs of which more than 400 are directly attributed to yeasts [1]. Despite wines containing such a complex array of VOCs, the most important aroma impact compounds yeast produces during fermentation are higher alcohols, acetate esters, and fatty acid esters [2]. Many metabolites, including VOCs are catabolically and anabolically formed via numerous interconnected metabolic pathways, which are metabolically, allosterically regulated via co-valent modification of enzymes, and are yeast strain-dependent [3–6]. Because of the strain-to-strain differences and the complexity of the regulation of metabolism of these aroma impact molecules, quite a lot is still not understood about the metabolism, making control of VOC production through processing changes or hard-wired genetic differences difficult. Therefore, to optimize and improve the production of wines, a more profound understanding of the metabolism of commercial yeast strains and their metabolic differences is required.

Currently, many commercial wine yeast strains have been reasonably well characterized on a phenotypic, biochemical, and even genotypic level [7]. Unfortunately, the relationships between a finished wine’s aroma characteristics and the microbial culture conditions that synthesize its bouquet are extraordinarily complex. However, with the rapid advent of new technologies and tools such as genome-scale metabolic models (GSMMs) [8], Constraint-Based Modeling (CBM) techniques can offer insight into yeast metabolism that will lead to the implementation of knowledge based changes in processing conditions or the introduction of novel commercial strains to achieve stylistic goals.

The production of VOCs such as esters and higher alcohols has been linked to the nitrogen requirements of yeast strains [9, 10]. Furthermore, commercial yeast strains regulate biomass and ferment at different rates and their nutrient utilization varies among strains [11]. Generally, commercial yeast strains have a higher nitrogen utilization efficiency (NUE) than laboratory yeast strains. NUE and the formation of VOCs are known to be correlated [12], where strains with a high utilization of nitrogen have been found to produce more esters and fewer higher alcohols. However, the metabolic mechanism to explain this connection has yet to be fully explored. Since aroma and flavor are central quality features of wines, many studies have been conducted to better understand the effects of juice nutrients and yeast choice on the final aroma profile [10, 12–14]. Despite these studies, it would be highly insightful to go beyond mere correlations to explore which metabolic pathways are involved in strain-specific VOC phenotypes.

Within the species, *S. cerevisiae* and other members of the genus *Saccharomyces*, the production of many VOCs is known to be strain-dependent [3, 15]. Although all wine yeast strains produce many similar aroma compounds, yeast genetics and physiology govern the production of esters, fatty acids and higher alcohols [5, 16], H_2_S formation [17–19], and volatile thiol release and conversion [20, 21]. Moreover, despite some relative success in properly overexpressing alcohol dehydrogenases (ADHs) and deleting some transaminases (BATs) contained within the Ehrlich pathway to steer higher alcohol formation [22–24], simple mutations can lead to inconclusive results or undesired effects such as overexpressing ARO9 which could cause unwanted overproduction of some higher alcohols [25]. These results highlight the need for modeling tools to globally examine the complex and intricate metabolic routes taken by yeast to produce various aromas. Over the past two decades, many GSMMs of *S. cerevisiae* have been produced, and validated by incorporating information from high-throughput omics data sets [26–30]. Some GSMMs have already been applied to examine yeast metabolism and improved the production of several commodity chemicals. For example, a genome-scale model referred to as, iFF708, has been used in a broad array of strain design applications ranging from enhancing biofuel production to optimizing succinate yields [31–33]. In addition, flux balance analysis (FBA) has been applied to a GSMM, iND750, to efficiently steer fumaric acid formation in *S. cerevisiae* [34]. Despite these groundbreaking efforts paving the way for applying GSMMs to steer yeast cell factories, these works are not directly applicable to enological fermentations because of the yeast strains modeled, the system being carbon-limited, and/or the system being aerobic.

While several studies have successfully modeled yeast under enological conditions from simple kinetic models [35] to genome-scale dynamic FBA (dFBA) models [36], they are limited in terms of describing the behavior of metabolites that contribute to organoleptic wine properties. Additionally, secondary metabolism is highly involved, and these initial models that focused on nitrogen metabolism did not contain information regarding the genes responsible for this association. Recent development and expansion in the latest yeast GSMM have allowed for a more significant investigation into pathways responsible for VOC formation [8, 30]. However, employing conventional CBM methods such as FBA and flux variability analysis (FVA) can be inadequate due to relying on a singular objective such as maximizing biomass. A powerful alternative approach, known as Monte Carlo random flux sampling, which has been applied to several GSMMs [28, 37, 38], provides a way to analyze genome-scale networks without needing an objective function. Flux sampling has the added benefit that it determines the feasible solution spaces for fluxes in a network based on a set of conditions as well as the probability of obtaining a solution [39]. Given the immense number of reactions involved in linking amino acid degradation and other nutrient utilization pathways to the formation of VOCs, CBM techniques provide a suitable option to further examine this relationship. Moreover, flux sampling presents a tool that could enable a comprehensive understanding of the flux solution space and the interrelationship between aroma-associated pathways of various strains at different stages of growth without specifying an objective function, especially when extensive data sets for multiple strains are available.

In this study, experimental data for four commercial yeast strains with varied VOC production patterns were used to calculate external fluxes of nutrients and VOCs throughout the fermentation. Here, a flux sampling approach was applied using the most recent genome-scale model of yeast metabolism, Yeast 8.4.2, to systematically determine how extracellular metabolite level fluctuations are related to comprehensive changes in intracellular metabolic flux states. Using flux sampling and statistical methods, intracellular metabolic conditions were successfully characterized without specifying a single optimal flux state as previously demonstrated [28, 38, 40]. Furthermore, by applying flux sampling, the metabolic states were compared at different stages of cell growth. Not only were these fluxes able to be evaluated at different time intervals, but four commercial wine yeast strains were compared to examine their key metabolic differences for diverse phenotypes. Lastly, probable genetic divergence was assessed among the strains by examining overlapping abundance of usage of notable gene associated reactions.

## Results

### Extracellular fluxes at various growth phases

**Fig. 1:**
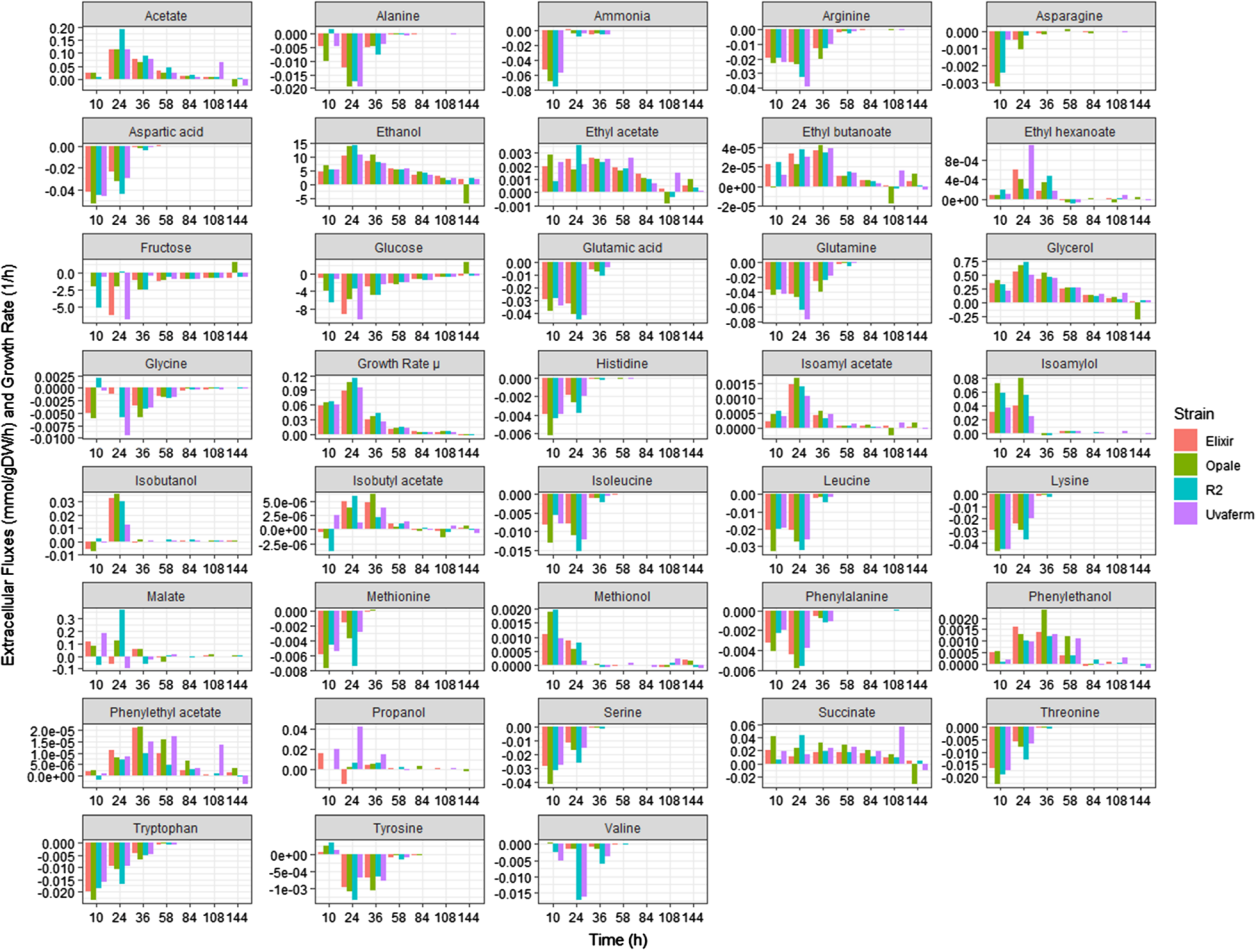
Bar chart of the fluxes used as constraints for the Monte Carlo sampling analysis

Extracellular fluxes of key primary and secondary metabolites, as well as specific growth rates of four yeast strains at various phases of cell growth, were derived from previously obtained experimental fermentation data [15]. Subsequently, it was examined the derived extracellular fluxes at multiple stages of growth and observed that most of the rapid formation of VOCs, especially fusel alcohols, coincided in time with the greatest rates of consumption of nitrogenous compounds and highest specific growth rates (Fig. 1). These phenomena all took place during the exponential growth phase, which was before 36 h after the start of the fermentation. The findings here support those found in previous studies [15] which showed the maximum production rate of many fusel alcohols and other aroma precursors occurs during the exponential growth phase. Some of the VOCs maintained relatively steady production during deceleration (pre-stationary) growth phase (until 58 h), including 2-phenyl ethanol, 2-phenylethyl acetate, and isobutyl acetate. This could be linked to the later consumption of nutrients such as tryptophan and tyrosine. Interestingly, a few VOCs, e.g., ethyl acetate, and ethyl butanoate, sustained moderate production rates well into the stationary growth phase. Overall, this could suggest other nutrients govern the production of these particular VOCs. Acetate and ethanol, in addition, could also play a role in later phases of VOC formation. Since it was concluded from statistical analysis that nutrient consumption was similar across strains, there were most likely underlying intracellular (metabolic) flux differences among the strains causing the variations in VOCs levels across the strains.

### Principal component analysis of extracellular fluxes

**Fig. 2:**
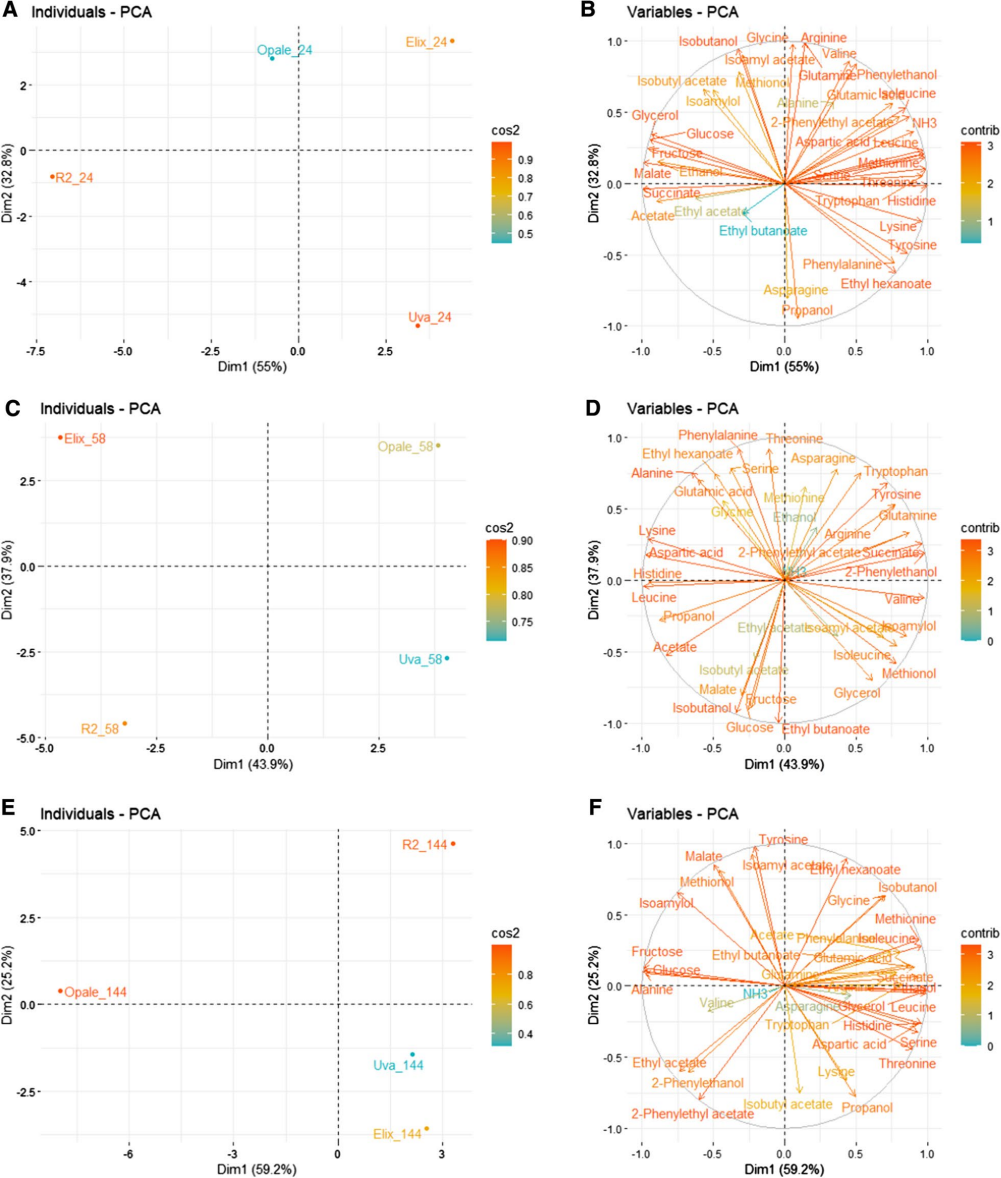
PCA results for the four yeast strains. Panels **A**, **C**, and **E** are individual factor maps at 24 h, 58 h, and 144 h, respectively. Panels **B**, **D**, and **F** are variable factor maps showing the effect of constraint fluxes significant for the PCA at 24 h, 58 h, and 144 h, respectively. The color scale is based on the cos2 value of each flux where the higher squared (cos2) loading values indicate greater importance in the PCA

To compare the extracellular fluxes across the strains, principal component analysis (PCA) was used to analyze fluxes relative to the stage of fermentation. (Fig. 2). From the PCA at 24 h, 87.8 % of the variance was explained by the first two principal components (PC) (PC1 = 55 % and PC2 = 32.8 %). The PCA at 58 h indicated a variance of 81.8 % was explained two PCs (PC1 = 43.9 % and PC2 = 37.9 %). Moreover, from the PCA at 144 h, 84.4 % of the variance was explained by the first two PCs (PC1 = 59.2 % and PC2 = 25.2 %). As depicted, separation of the samples was achieved according to the yeast strains (Fig. 2A, C). For the fluxes at 24 h, PC1 separated Uvaferm and Elixir from the other two strains, while PC2 separated Opale and Elixir from the other strains (Fig. 2A). The strains were neatly separated from each other for the fluxes at 58 h where each strain was contained in their own quadrant (Fig. 2C). For the fluxes at 144 h, PC1 separated Uvaferm and Elixir from the other two strains while PC2 separated R2 from the other strains (Fig. 2E). It is notable that R2 remained the distinct strain revealed by the PCA at the two time points. Furthermore, variation is illustrated between the metabolisms of four yeast strains at different fermentation phases.

In order to reveal the important fluxes that drive the variation in different time points (24, 58 and 144 h), a variable factor map was plotted, and these variables are shown with a color scale based on their cos2 values (Fig. 2B, D, F). Several of the important variables for each phase are illustrated (Fig. 2). Uvaferm at 24 h was most associated with propanol and ethyl hexanoate. R2 was most associated with ethyl acetate while Elixir was most associated with 2 phenylethanol and 2 phenylethyl acetate. Opale had a low cos2 value, but it was associated with isobutanol and isoamyl acetate. Accordingly, several extracellular fluxes related to amino acid utilization were positively associated with Elixir, Uvaferm, and R2 strains at this stage of fermentation. Here, at 24 h it was observed for Uvaferm strong contributions were present from asparagine, phenylalanine, and tyrosine (Fig. 2B). Elixir contained most contributions from valine, leucine, and isoleucine whereas Opale was most associated with glycine and alanine. R2 stoodout in that it was most associated with fluxes from carbon metabolism at 24 h. These fluxes were succinate and acetate (Fig. 2B).

The variable factor map for 58 h illustrates changes in carbon and amino acid substrate variables associated with the yeast strains (Fig. 2D). Changes were most prominently noticed in variables associated with glucose, fructose, glycerol, isoleucine, valine, lysine, and phenylalanine. More specifically, R2 at 58 h is most associated with glucose and isobutanol (Fig. 2D). Opale at 58 h is most associated with asparagine and tryptophan. However, Elixir is most associated with phenylalanine and serine while Uvaferm is most associated with glycerol and isoleucine.

At 144 h of fermentation, many VOCs were not produced as they were at 24 h. The variable factor map for 144 h illustrated shifts in VOC variables associated with the yeast strains (Fig. 2F). For instance, R2 at 144 h was most associated with ethyl hexanoate and isobutanol (Fig. 2F). Opale at 144 h was most associated with isoamylol whereas Uvaferm and Elixir were most associated with propanol and isobutyl acetate. The variable associations and patterns noticed at the 24 h, 58 h, and 144 h time points from PCA suggested strain-specific influences from metabolism could be promoting distinct VOC character among the strains. Taken together, the separation of the strains is associated to different metabolic features (metabolites) at each time point, suggesting general strain variation.

### Examining strain-specific metabolic differences using flux sampling

Flux sampling was applied using Yeast 8.4.2 and constrained using experimental flux data at several times during yeast cell growth (24, 58, and 144 h) (Fig. 1) to evaluate the metabolic changes as it pertains to the differences in VOCs formation. The converged flux sampling distributions were computed for all model reactions. In order to discern and establish which network fluxes contribute the most to the phenotypic differences in the yeast strains, all of the reactions in the network were examined, but analysis focused on the top 20 reactions based on their absolute differences in simulated medians of the sample distribution values for the four strains. The top 20 reactions were also evaluated based on their percent differences in simulated median of the sample distribution values at several times during yeast cell growth (24, 58, and 144 h). However, many of these reactions contained miniscule fluxes relative to the VOC exchange flux values (< l0^−12^ mmol/ (gDW h)). The top 20 reactions based on absolute differences are listed in Table 1.

**Table 1:** Summary of the top 20 reactions based on absolute differences in flux medians at 24 h among the yeast strains, and their corresponding gene associations and metabolic subsystems. Reactions are listed according to absolute median differences starting with the largest

Genes (Short Name)	Gene Association	Reaction Names	GSMM Reaction Number	Metabolic Subsystem
ARO9	YHR137W	Tyrosine transaminase	r_2119	Tyrosine metabolism, Biosynthesis of secondary metabolites (Ehrlich pathway)
ALT2	YDR111C	L-Alanine:2-oxoglutarate aminotransferase	r_4226	Alanine metabolism
ACO2	YJL200C	Citrate hydroxymutase	r_4262	Citric Acid Cycle
ADH5 ADH1	YBR145W YOL086C	Alcohol dehydrogenase, (acetaldehyde to ethanol)	r_2115	Glycolysis, Fatty acid degradation, Tyrosine metabolism, Biosynthesis of secondary metabolites (Ehrlich pathway)
GLO2	YDR272W	Hydroxyacylglutathione hydrolase	r_0553	Pyruvate metabolism
GLO1	YML004C	Lactoylglutathione lyase	r_0697	Pyruvate metabolism
HSP31 SNO4 HSP33 HSP32	YDR533C YMR322C YOR391C YPL280W	(R)-lactate hydro-lyase	r_4236	Other carbon metabolism
PGK1	YCR012W	Phosphoglycerate kinase	r_0892	Glycolysis, Carbon metabolism
TDH3 TDH1 TDH2	YGR192C YJL052W YJR009C	Glyceraldehyde-3-phosphate dehydrogenase	r_0486	Glycolysis, Gluconeogenesis, Carbon metabolism, Biosynthesis of secondary metabolites
CDC19 PYK2	YAL038W YOR347C	Pyruvate kinase	r_0962	Pyruvate metabolism, Glycolysis, Purine metabolism, Carbon metabolism,
GPM1	YOR283W YKL152C	Phosphoglycerate mutase	r_0893	Glycine, serine and threonine metabolism, Glycolysis, Carbon metabolism
PDC6 PDC1 PDC5	YGR087C YLR044C YLR134W	Pyruvate decarboxylase	r_0959	Glycolysis, Gluconeogenesis, Biosynthesis of secondary metabolites (Ehrlich pathway)
ADH2	YMR303C	Alcohol dehydrogenase (ethanol to acetaldehyde)	r_0163	Glycolysis, Tyrosine metabolism, Biosynthesis of secondary metabolites (Ehrlich pathway), Fatty acid degradation
GLK1 HXK1 HXK2 EMI2	YLR446W YCL040W YFR053C YGL253W YDR516C	Hexokinase (D-glucose:ATP)	r_0534	Glycolysis, Gluconeogenesis, Fructose and mannose metabolism, Galactose metabolism, Amino sugar and nucleotide sugar metabolism, Carbon metabolism, Biosynthesis of secondary metabolites
AAT2	YLR027C	Aspartate transaminase	r_0216	Alanine, aspartate and glutamate metabolism, Tyrosine metabolism, Cysteine and methionine metabolism
HOM2	YDR158W	Aspartate-semialdehyde dehydrogenase	r_0219	Glycine, serine and threonine metabolism, Cysteine and methionine metabolism
HOM6	YJR139C	Homoserine dehydrogenase (NADH)	r_0546	Glycine, serine and threonine metabolism; Cysteine and methionine metabolism, Biosynthesis of secondary metabolites
THI3 PDC6 PDC1 PDC5	YDL080C YGR087C YLR044C YLR134W	3-methyl-2-oxopentanoate decarboxylase	r_0064	Glycolysis, Gluconeogenesis, Biosynthesis of secondary metabolites (Ehrlich pathway)
GPD1 GPD2	YDL022W YOL059W	Glycerol-3-phosphate dehydrogenase (NAD)	r_0491	Glycerophospholipid metabolism, Biosynthesis of secondary metabolites
GND1 GND2	YGR256W YHR183W	Phosphogluconate dehydrogenase	r_0889	Glutathione metabolism, Carbon metabolism, Biosynthesis of secondary metabolites

**Fig. 3:**
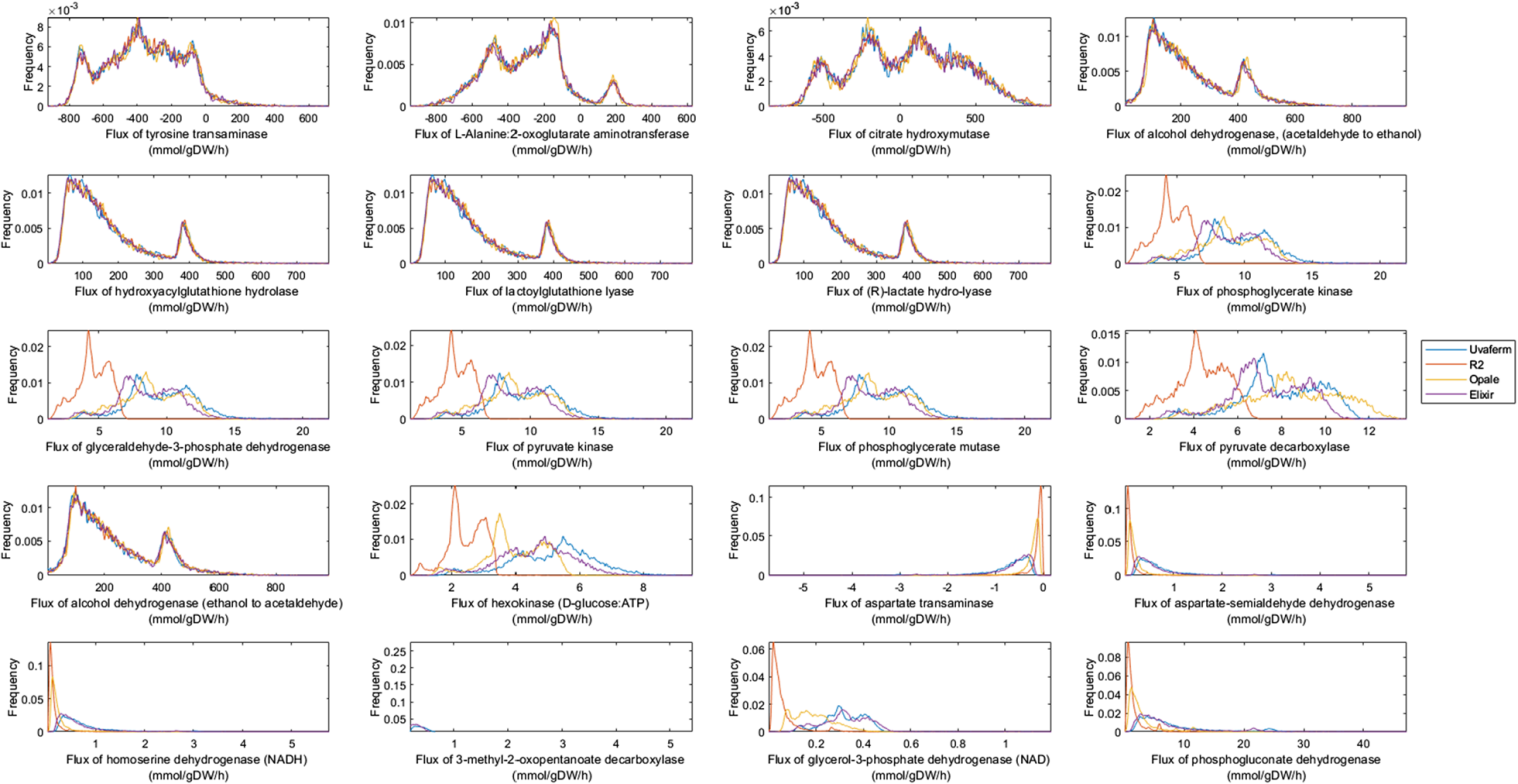
Comparison of four phenotypes: Uniform random sampling plots of relative frequency vs. predicted flux of key reactions linked to aroma formation for four strains - Uvaferm, R2, Opale, and Elixir during the exponential growth phase (24 h.)

Random flux sampling was first performed to assess metabolic flux distribution differences among the strains during the exponential growth phase. Next, random sampling histograms were compared among the top 20 fluxes that relate to phenotypic strain variation (Fig. 3). Here, broad flux distributions as well as relative similarities were seen in flux magnitudes among the strains for most of the transaminase reactions except aspartate transaminase (r_0216). However, some variation was noticed, particularly with the R2 strain regarding glycolysis and some other central carbon metabolism-associated reactions (r_0892, r_0486, r_0962, r_0893, and r_0534). Furthermore, there was some noteworthy characteristic separation in the distributions among all of the strains related to a known aroma-associated reaction: r_0959 - pyruvate decarboxylase. There were similar attributes regarding amino acid dehydrogenases (r_0219 and r_0546). It was noticed that variation was divided among the strains where Elixir and Uvaferm are grouped together, and the other two strains are not.

**Fig. 4:**
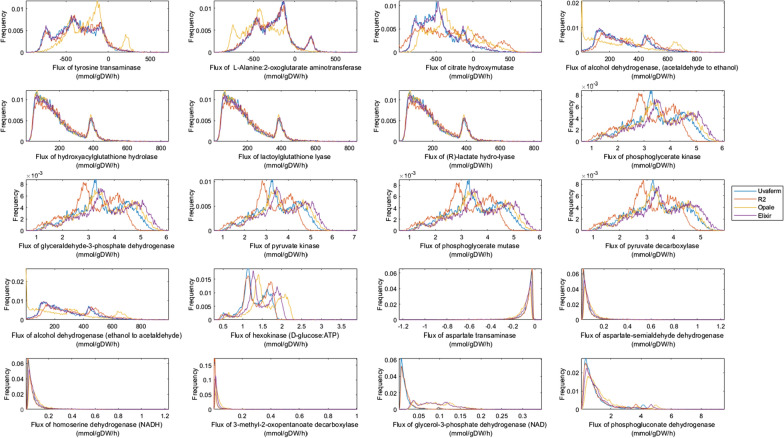
Comparison of four phenotypes: Uniform random sampling plots of relative frequency vs. predicted flux of key reactions linked to aroma formation for four strains - Uvaferm, R2, Opale, and Elixir during the deceleration phase (58 h.)

Using flux sampling, the metabolic solution space was then explored among the strains during the deceleration phase to understand how yeast metabolism changes throughout fermentation. Here, it was noticed that many of the transaminase reactions have distribution patterns similar to those during the exponential growth phase, but the Opale strain shows distinct distributions among the strains (Fig. 4). The citrate hydroxymutase solution distributions were chiefly varied among Opale and R2 strains. For the alcohol dehydrogenase reaction (r_0163), the Opale strain was distinctive from the other strains indicating flux differences to produce higher alcohols. For amino acid dehydrogenases (r_0219 and r_0546) as well as an aspartate transaminase and 3-methyl-2-oxopentanoate decarboxylase (r_0216 and r_0064), the flux distributions were narrower and closer to a certain flux value compared to during the exponential phase. There was also greater similarity among the strains for these reactions. However, for the glycerol-3-phosphate dehydrogenase reaction the flux distributions were shown to separate the Uvaferm and Elixir, and Opale and R2 groups. Interestingly, when examining the central carbon metabolism-associated reactions (r_0892, r_0486, r_0962, r_0893, r_0889, and r_0534), an increasing disparity in flux distributions became apparent. In contrast, for other related metabolic reactions (r_0553, r_0697, and r_4236), the distributions remained characteristically unchanged going from exponential to deceleration growth phase (Fig. 4).

**Fig. 5:**
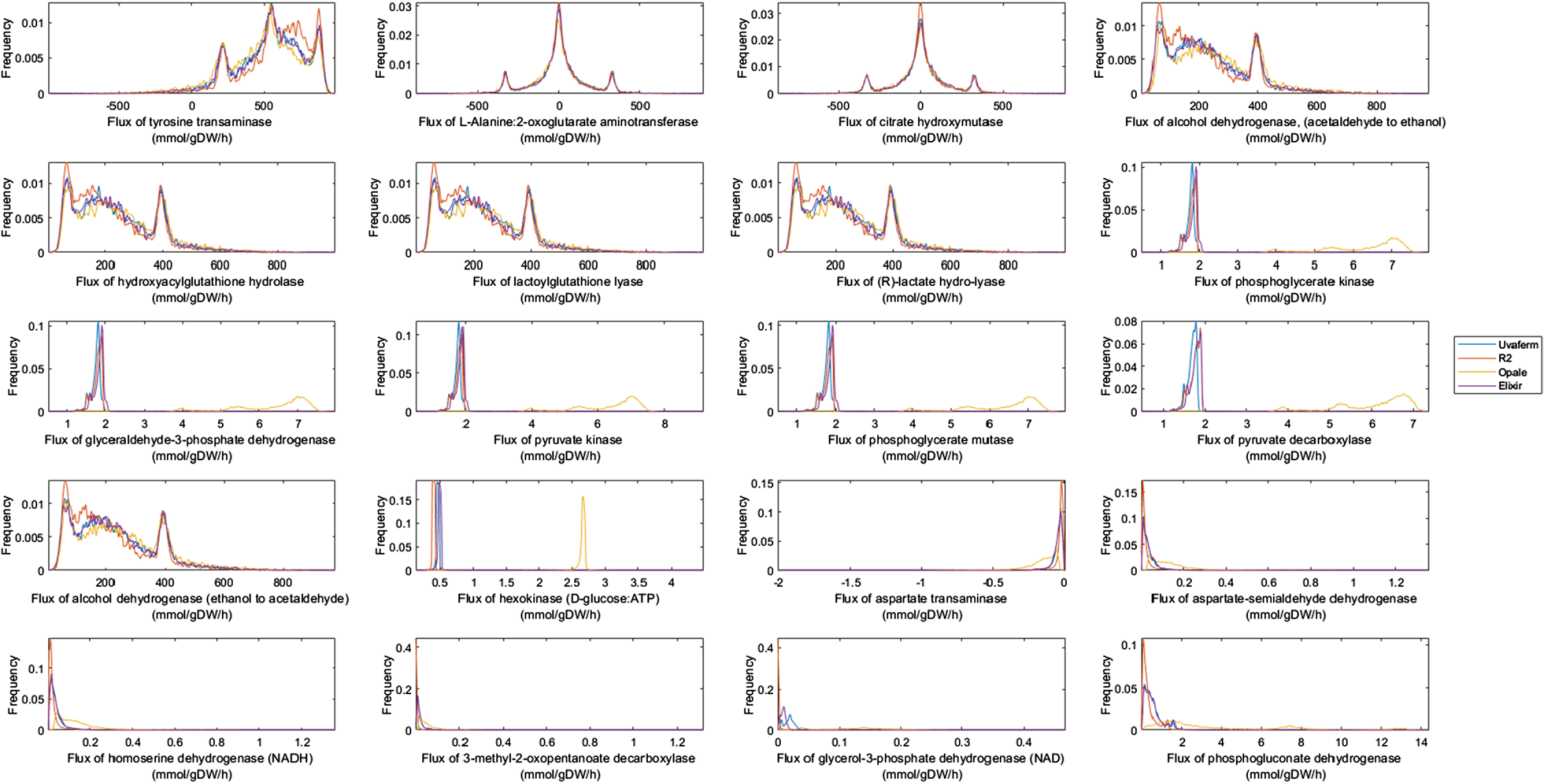
Comparison of four phenotypes: Uniform random sampling plots of relative frequency vs. predicted flux of key reactions linked to aroma formation for four strains - Uvaferm, R2, Opale, and Elixir stationary phase (144 h.)

During the stationary phase, it was apparent from flux sampling that the flux distributions of many of the top 20 fluxes shifted to narrower, more centered distributions where the Opale yeast strain was the predominant outlier among the strains (Fig. 5). The first four presented fluxes (Fig. 5) in comparison to other growth phases experienced similarly broad flux distributions, which ranged from negative to positive values centered at relative fluxes values. The hydroxyacylglutathione hydrolase, lactoylglutathione lyase, and the (R)-lactate hydro-lyase associated reactions (r_0553, r_0697, and r_4236) exhibited nearly identical characteristic flux distributions throughout all of the examined phases of growth. It was striking that the alcohol dehydrogenase-associated reaction (r_2115) had a distinguished distribution during pre-stationary phase growth from the exponential phase yet reverted to a similar distribution as the exponential phase during the stationary growth phase. Moreover, the central carbon metabolism-associated reactions (r_0892, r_0486, r_0962, r_0893, and r_0534) shifted immensely from the various growth phases until all of the strains, except Opale, converged to having identical flux distributions during the stationary growth phase (Fig. 5). The strain similarity trend continued when observing other reaction distributions (r_0219, r_0546, r_0216, and r_0064) where Opale stood out among the strains. This characteristic was especially glaring when looking at hexokinase associated reaction (r_0534).

### Cluster analysis comparison of the yeast strains

**Fig. 6:**
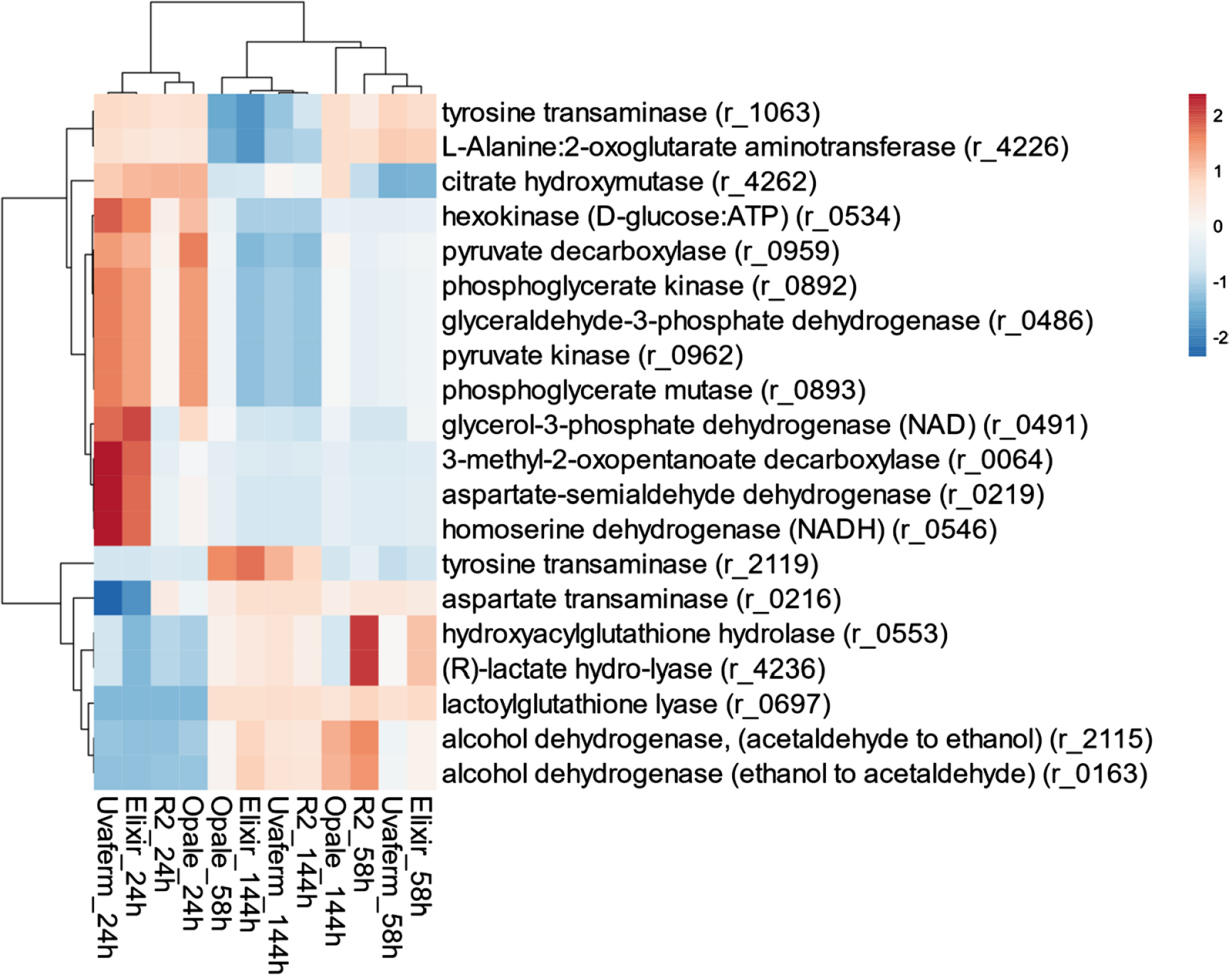
Hierarchical clustergram depicting from the flux sampling analysis how alike/different the strains are regarding top 20 absolute reactions to infer gene associations. The correlation bar on the upper right is based on Pearson correlations

In order to assess how the yeast strains are related to each other, a hierarchical clustergram was generated for the model predictions based on metabolic gene association or phenotype predictions (see Materials and Methods). The clustergrams were constructed from the median values of the flux sampling analysis of the top 20 absolute different reactions (Fig. 6). This was done as a proxy to qualitatively gauge the relative genotype of each yeast strain using gene-protein reaction (GPR) associations. From the cluster analysis, Elixir and Uvaferm were found to be the most similar to one another. Then, Opale was determined to be relatively similar to the Elixir and Uvaferm pair. Lastly, R2 strain was indicated to be the least like the other yeast strains. Remarkably, it was observed when investigating the differences among the strains using the flux sampling, the results from all of the reactions known to be aroma-associated such as ones related to amino acid degradation, see Scott et al. [15], the clustering order shifted among the strains (Fig. 6). Although Elixir and Uvaferm strains were also the most alike among the strains when looking at aroma-associated reactions, Opale appeaed to be the most distinct strain. In other words, when just examining reactions known to be associated with VOC formation such as Ehrlich pathway and lipid degradation pathways, the differences remained consistent with the top 20 reactions. Moreover, R2 and Opale were still shown to be the most distinct strains. It is interesting to point out that this result contradicts the manufacturer’s description of Uvaferm being a neutral aroma producing yeast strain while the other strains are regarded as imparting specific aroma attributes to wines e.g., producing more esters or a certain combination of VOCs. As it was observed at 24 h, decarboxylase and dehydrogenase reactions related to amino acid degradation as well as glycerol dehydrogenase reactions from central carbon metabolism were most different among the strains. While on the other hand at 58 h, most of the variation among the strains was attributed to reactions associated with pyruvate and other carbon metabolism.

## Discussion

In this work, a CBM of random flux sampling was used to examine the differences in intracellular metabolic flux states of commercial wine yeasts under typical enological fermentation conditions. The fluxes were derived from experimental measurements of numerous nutrients, including amino acids and sugars, as well as primary and secondary products, including key VOCs at different phases of cell growth. They were used to constrain the model for flux sampling analysis. The intracellular metabolic flux states were successfully characterized without the need of designating an objective function for optimal states as was necessary in earlier works [28, 38, 40]. However, this work is novel, in that it applies established flux sampling and statistical approaches to understand the underlying differences in metabolism among commercial wine yeasts and, thus, why these strains produce distinct aromas based on those metabolic differences. By choosing to examine metabolism globally, and then focusing on the top 20 absolute fluxes that pertain to the greatest absolute differences from flux sampling, VOC differences could be corroborated which were demonstrated from PCA results with intracellular differences in metabolic states. In particular, this is novel because we examined the parts of yeast metabolism most responsible for strain-specific aroma behavior exhibited by commercial wine yeast strains. Furthermore, our genome-scale modeling work highlights the intricate roles carbon, nitrogen, and lipid metabolism of yeast play in producing VOCs, as shown experimentally in other studies [10, 12–14, 41, 42].

While several earlier studies have focused on integrating extracellular metabolite concentration or flux measurements with yeast GSMMs, those studies pertained to nutrient-rich media or aerobic processes [28, 43, 44]. Additionally, although studies have employed CBM approaches to yeast GSMM under enological conditions, they used flux balance analysis or relied on optimization routines to obtain predictions of metabolic flux states [8, 36]. Furthermore, these studies relied on biased optimization strategies without also exploring the entirety of solution space throughout different growth phases. However, research has demonstrated how random flux sampling can analyze metabolic differences across multiple conditions while eliminating the need for assuming an optimal flux state [28, 38, 45]. The main disadvantage of using a random sampling approach is that there is a link missing between the fluxes for a particular solution. Also, the modes of each distribution are assumed to create an overall feasible solution. This is impossible when observing modes of each distribution. Despite this disadvantage, the metabolic flux solution space of various phenotypes can still be qualitatively compared and analyzed. Nearly all of the previous works that studied or applied yeast GSMMs did not use yeast GSMMs with a detailed set of peripheral metabolic reactions such as extended (Ehrlich) amino acid degradation and sulfur pathways known to be associated with VOCs [2, 46, 47] or lipid biosynthesis pathways that might play an essential role in protecting yeast cell membrane against ethanol toxicity, enhancing growth, and producing VOCs [42, 48]. In this study, those shortcomings of previous studies were addressed by using flux sampling, an unbiased modeling approach, to investigate primary and secondary yeast metabolism at various growth phases.

Opale yeast was associated with isobutanol, isoamylol, and isoamyl acetate, therefore it makes sense that it was higher in median flux of the pyruvate decarboxylase reaction (r_0959) because this reaction leads to isoamylaldehyde which is a precursor for isoamylol and isoamyl acetate. On the other hand, R2 which was lower in all VOCs, has higher median fluxes going toward central metabolism (see reactions r_0892, r_0486, and r_0962) and a lower median flux in the reaction associated with glycerol-3-phosphate dehydrogenase (NAD) (r_0491) which is related to the synthesis of secondary metabolites (VOCs). The other two strains, Elixir and Uvaferm, were associated with 2-phenylethanol, propanol, and ethyl hexanoate. Therefore, it is reasonable that the median fluxes for reactions r_0216, r_0219, r_0064, and r_0491 were higher as these reactions lead to the precursors for these VOCs. Since these two strains were lower in isoamylol and isobutanol, it makes sense that the median flux for the hexokinase associated reaction (r_0534) was higher than that for Opale.

In this work, it was observed that Uvaferm and Elixir strains behaved similarly while Opale and R2 were the most distinct. The results point to amino acid and pyruvate metabolism being more active in Opale. Therefore, Opale is associated with isoamylol, isobutanol, and isoamyl acetate. R2 was shown to have lower activity in amino acid and pyruvate metabolism, and hence has lower levels of VOCs than the other strains. Uvaferm and Elixir are similar to each other when examining central carbon, pyruvate, amino acid, and fatty acid degradation metabolism where we predicted higher median fluxes. That would explain why Uvaferm and Elixir produce higher amounts of 2-phenylethyl acetate and ethyl hexanoate, and lower amounts of ethyl acetate. Overall, the phenotypic differences among the strains are predicted to stem from major differences in pyruvate, tyrosine, glycine, serine, threonine and central carbon metabolism. Random flux sampling also predicted substantial differences in metabolic pathways responsible for the generation of secondary metabolites such as Ehrlich pathway. Using Yeast 8.4.2 coupled with our flux sampling approach allowed us to compare predicted fluxes of relevant pathways at different growth phases among the commercial yeast strains. Moreover, by using genome-scale CBM, some insight could be gathered into the global, interconnected pathways responsible for the variation in metabolism and, thus, aroma-producing capabilities among the strains. This work indicates central carbon, amino acid, sulfur, and lipid metabolism play varying roles throughout fermentation to lead to strain-specific characteristics. Results highlighted here reveal the need for more studies to comprehensively investigate nitrogen and lipid metabolism as well as central carbon metabolism to understand their impact on yeast aroma formation. For instance, studies have confirmed the essential role acetaldehyde has within core carbon metabolism and have linked the enzyme ADH2 to oxidizing ethanol to form acetaldehyde [49]. Subsequently, to regulate the amounts of acetaldehyde and limit the production of acetic acid, which forms from acetaldehyde oxidation, ADH2 modulation has been performed in yeast resulting in an 82 % reduction of acetaldehyde [50].

From the flux sampling analysis, it was observed that not only ADH2 associated reactions are accounted for the strain variation, but also PDC1, PDC5, and PDC6 pyruvate decarboxylase as well as 3-methyl-2-oxopentanoate decarboxylase related reactions. This result highlights the interdependent relationship of carbon and nitrogen metabolism and how the expression of intricate pathways can lead to aroma differences. PDC1, PDC5, and PDC6 are involved in the Ehrlich pathway, leading to the irreversible decarboxylation of the α-keto acid to an aldehyde [47]. This pathway linkage between PDCs and ADHs has been exploited in attempting to drive the production of higher alcohols. More specifically, paired with deletion of BAT1 (transaminase) and ALD6 (the aldehyde dehydrogenase) plus overexpression of ARO10 and ADH2, Park and coworkers were able to steer higher alcohol formation [24]. Interestingly, reaction fluxes associated with Ehrlich pathway reactions such as transaminases, decarboxylases, and alcohol dehydrogenases were found to be related to critical metabolic differences among the strains. Conversely, many reaction fluxes were associated with other metabolic pathways pointing to the need for further study to understand the strain-specific behavior.

## Conclusions

The CBM approach utilized in this work analyzed and compared the various predicted intracellular metabolic flux states of commercial yeast strains during enological fermentation, including examining the metabolic shifts within the production of VOCs and the consumption of nutrients (amino acid, sugars, and ammonium). The intracellular flux distribution predictions show qualitative agreement with the specific variations found from performing principal component analysis on extracellular flux values. Furthermore, these results indicate elaborate fluctuations and distinctness in nitrogen, carbon, and lipid metabolism that lead to strain-specific differences in VOC formation. From the changing metabolic flux distributions among the strains, the differences in GPR activity were compared and highlighted. Therefore, probable genetic differences among the strains could be inferred and targets for genetic modification could be explored. Although the results in this study identified nitrogen metabolism which is supported by other works as causing VOC specific strain behavior, the results also show carbon and lipid metabolism play a role in VOC formation. This revelation points to the need for additional studies to explore impact of other parts of metabolism on VOC formation in yeasts. Overall, the approach and insight gained here were in good agreement with experimental observations and other studies, making this a promising approach for future use in studies related to individual fluxes of important metabolites in enological conditions and comparing metabolic differences between commercial wine yeast strains. In addition, this work help spur new quests in creating more precise aroma producing wine yeast strains.

## Materials and methods

### Experimental data

The experimental data used in this study is from Scott et al. [15]. In this work, this dataset was used to apply CBM approaches. Moreover, specific consumption and production rates (fluxes) were estimated from the experimentally measured compounds presented in Scott et al.[15] at various time points throughout the fermentation.

The yeast strains used in experiments were Uvaferm 43^TM^(Uvaferm), Lalvin R2^TM^ (R2), Lalvin ICV Opale^TM^ (Opale), and Vitilevure^TM^ Elixir YSEO (Elixir). All strains were Lallemand (Lallemand, Montreal, Quebec) commercial yeast strains. In addition, all yeast strains were obtained from the UC Davis Enology Culture Collection containing the following culture collection numbers: Uvaferm (UCD4004), R2 (UCD2033), Opale (UCD2797), and Elixir (UCD4008). These yeast strains were selected based on the different fermentation and aroma-producing performance attributes reported by the manufacturer.

### Genome-scale metabolic model

The GSMM employed in this study was *Yeast 8.4.2* [30], which is widely available via GitHub. Overall, the GSMM contains 2742 metabolites, 4058 reactions, and 1150 genes. The GSMM is designed for *S. cerevisiae*, S288C, a laboratory yeast strain not typically used in industrial settings. However, since this study was applied to fermentations under enological conditions, the GSMM was modified to reflect the anaerobic state of metabolism appropriately. Here, a strategy was applied as described by Heavner et al. [51], constraining ${v}_{{O}_{2}}$ to zero (LB=UB=0 [mmol/(g DW h)]), allowing unrestricted uptake of ergosterol (r_1757), lanosterol (r_1915), zymosterol (r_2106), 14-demethyllanosterol (r_2134), ergosta-5,7,22,24(28)-tetraen-3beta-ol (r_2137), and oleate (r_2189). In addition, pathways including the oxaloacetate-malate shuttle and glycerol dehydrogenase reaction were unrestricted as described by Sanchez et al. [52, 53] (in the model this was achieved by blocking reactions r_0713, r_0714, and r_0487). Heme A was also removed from the biomass equation as it is not used under anaerobic conditions. Moreover, *Yeast 8.4.2* includes expanded coverage of aroma-associated pathways such as an extended Ehrlich pathway, more ester formation reactions, and enhanced sulfur reduction pathways as previously performed and described in the literature [8].

### Model constraints

The experimentally measured net uptake and production fluxes (see Fig. 1) were applied as experimental constraints in the form of flux bounds that restrict the uptake and product fluxes in the model. More specifically, exchange (i.e. transport) reactions for the sugars, amino acids, organic acids, VOCs and other byproducts were set according to flux values (LB=UB) from a chemically defined medium during anaerobic nitrogen-limited fermentation data found in the literature [15]. The experimental fluxes used as constraints were derived from concentration vs. time datasets with numerical derivatives estimated by employing a finite difference method (Euler’s method). The finite difference method involved using concentrations values at both sides of a time point (midpoint method) without prior smoothing. The derived production and secretion fluxes were then normalized by measured biomass concentrations.

### Statistical analysis

Data analysis was performed using R (version 3.6.2, R Core Team, 2020). (http://cran.r-project.org/). Principal component analysis (PCA) was conducted using the FactoMineR package [54]. Squared cosine (cos2) demonstrates the importance of a component for a given observation which is the vector of original variables. The squared cosine more specifically designates the contribution of a component to the squared distance of the observation to the origin. The hierarchical clustering heat map was generated using the Clustvis package in R [55]. The correlation bar was based on Pearson correlation coefficients.

### Monte Carlo random flux sampling

Random flux sampling is an adept approach used to characterize the solution space within a GSMM network. This method involves obtaining a statistically significant number of solutions that have been uniformly distributed throughout the entire solution space [56]. By using randomized flux sampling of candidate network states throughout an entire solution space, an unbiased assessment of its properties was obtained. The converged flux sampling distributions were computed for all model reactions. Flux sampling analysis was applied using optGpSampler [57], an efficient algorithm based on the Monte Carlo Artificially Centered Hit and Run (ACHR) [58] algorithm where the solution space - all possible flux states - are characterized using mass conservation and stoichiometric constraints (satisfying LB and UB constraints). The algorithm parameters were set for each experimental condition in order to sample 10,000 points and the limit was set to 1 × 10^10^ number of steps to reach a solution. The algorithm was employed to explore the distribution of solutions based on experimentally determined growth rates and the optimal flux range for each experimental condition. Therefore, the model aimed to characterize the solution space based on empirical growth rate and the corresponding observed consumption/production rates (see Fig. 1). To accomplish this, the upper and lower bounds of corresponding exchange reactions were fixed according to extracellular flux data (Fig. 1). Next, the algorithm was used for determining the flux distributions that were obtained based on our restrictions. The 20 reactions were found that represented the greatest absolute flux variations among the distributions among the yeast strains for every condition. However, the top 20 fluxes at 24 h were used, for instance, to compare at all times. The 20 reactions were also found that represented the greatest percent flux variation among the distributions among yeast strains for every condition (see Additional file 1: Table S1). However, it was concluded the top 20 reactions based on percent flux variation provides little metabolic insight as many of the identified reactions contain median fluxes that are miniscule relative to VOC exchange fluxes (< l0^−12^ mmol/ (gDW h)) and many were considered irrelevant because they are reactions describing transport between compartments. Finally, histograms were generated to characterize the solution space of the 20 key reactions, which contributed the metabolic difference among the strains related to experimental criteria. These histograms illustrated respective reaction fluxes along with solution frequencies. Random sampling was performed using Cobra Toolbox 3.0 [49] functions (see tutorial: https://github.com/opencobra/COBRA.tutorials/tree/master/analysis/uniformSampling).

### Computing environment

Modeling was performed in MATLAB® 2018b (The MathWorks, Inc., Cambridge, MA, USA) using Cobra Toolbox 3.0 [59] and implemented on a Windows 10 (Microsoft Corporation, Redmond, WA, USA) Intel® (Intel Corporation, Santa Clara, CA, USA) Core™ i7-7500 CPU @ 2.70 GHz–2.90 GHz processor. Git version 2.3.0 was installed before cloning COBRA with GitHub and initializing COBRA in MATLAB. The GSMM was imported into MATLAB, as an SBML file, and evaluated using Cobra Toolbox.

## Supplementary Information

**Additional file 1: Table S1**. The top 20 reactions that hat represented the greatest percent flux variation among the distributions among yeast strains for every condition.

## Data Availability

The datasets used and/or analyzed during the current study are available from the corresponding author on reasonable request.
