# Supplementary material for: Metabolic flux sampling predicts strain-dependent differences related to aroma production among commercial wine yeasts
Source: Microb Cell Fact. 2021 Oct 21;20:204. doi: 10.1186/s12934-021-01694-0 (PMC8532357; doi:10.1186/s12934-021-01694-0)
Supplement: Supplementary file 1 — Additional file 1: Table S1. The top 20 reactions that hat represented the greatest percent flux variation among the distributions among yeast strains for every condition. [file 12934_2021_1694_MOESM1_ESM.pdf]

**Table S1**. The top 20 reactions that hat represented the greatest percent flux variation among the distributions among yeast strains for every condition.

| Rxn ID | Rxn Names | Uvaferm Flux Median (1) | R2 Flux Median (2) | Opale Flux Median (3) | Elixir Flux Median (4) | abs( 1-2) | abs( 1-3) | abs( 1-4) | abs( 2-3) | abs( 2-4) | abs( 3-4) | **SUM diff** | percentage abs( 1-2) | percentage abs( 1-3) | percentage abs( 1-4) | percentage abs( 2-3) | percentage abs( 2-4) | percentage abs( 3-4) | **SUM percentage diff** | **rank based on summ perc diff** |
|---|---|---|---|---|---|---|---|---|---|---|---|---|---|---|---|---|---|---|---|---|
| r_4246 | 'Alpha-mannosidase (EC 3.2.1.24) (Alpha-D-mannoside mannohydrolase)' | 9.44E-16 | 6.04E-13 | 6.05E-13 | 6.10E-13 | 6.03E-13 | 6.04E-13 | 6.09E-13 | 1.1E-15 | 6.23E-15 | 5.13E-15 | 1.83E-12 | 638.8514 | 640.0115 | 638.8514 | 1.81E-03 | 1.03E-02 | 8.48E-03 | 1917.735 | 1 |
| r_0012 | '1-pyrroline-5-carboxylate dehydrogenase' | 1.56E-08 | 5.38E-11 | -8.86E-12 | 1.08E-08 | 1.55E-08 | 1.56E-08 | 4.78E-09 | 6.26E-11 | 1.07E-08 | 1.08E-08 | 5.75E-08 | 0.996548 | 1.000569 | 0.996548 | 1.16E+00 | 2.00E+02 | 1.22E+03 | 1423.742 | 2 |
| r_0490 | 'glycerol-3-phosphate dehydrogenase (fad)' | -9.3E-12 | 2.57E-09 | -1.2E-11 | -1.3E-11 | 2.58E-09 | 2.21E-12 | 3.92E-12 | 2.58E-09 | 2.58E-09 | 1.71E-12 | 7.76E-09 | 276.1259 | 0.236083 | 276.1259 | 1.004493 | 1.005159 | 0.148301 | 554.6458 | 3 |

| | | | | | | | | | | | | | | | | | | | | |
|---|---|---|---|---|---|---|---|---|---|---|---|---|---|---|---|---|---|---|---|---|
| r_3307 | 'DAG lipase (1-18:1, 2-18:1), mitochondrion' | -2E-15 | -3.16E-13 | -2.95E-13 | -3.05E-13 | 3.14E-13 | 2.93E-13 | 3.03E-13 | 2.1E-14 | 1.1E-14 | 1E-14 | 9.53E-13 | 160.8196 | 150.0489 | 160.8196 | 6.66E-02 | 3.49E-02 | 3.39E-02 | 471.8236 | 4 |
| r_4158 | 'NADPH2:quinone oxidoreductase' | -2.6E-15 | -2.58E-13 | -2.60E-13 | -2.60E-13 | 2.55E-13 | 2.57E-13 | 2.57E-13 | 2.03E-15 | 2.23E-15 | 2.07E-16 | 7.74E-13 | 97.38966 | 98.1635 | 97.38966 | 7.86E-03 | 8.67E-03 | 7.96E-04 | 292.9602 | 5 |
| r_4264 | 'succinate:NAD+ oxidoreductase' | -9.5E-13 | 1.30E-10 | -4.14E-12 | -9.83E-12 | 1.31E-10 | 3.19E-12 | 8.88E-12 | 1.34E-10 | 1.4E-10 | 5.69E-12 | 4.24E-10 | 137.8439 | 3.348375 | 137.8439 | 1.03E+00 | 1.08E+00 | 1.37E+00 | 282.518 | 6 |
| r_4296 | 'D-Glucuronolactone:NAD+ oxidoreductase' | 2.21E-15 | -1.92E-13 | -1.99E-13 | -1.98E-13 | 1.94E-13 | 2.02E-13 | 2E-13 | 7.35E-15 | 5.76E-15 | 1.59E-15 | 6.11E-13 | 88.08172 | 91.41335 | 88.08172 | 3.83E-02 | 3.00E-02 | 7.96E-03 | 267.653 | 7 |

| | | | | | | | | | | | | | | | | | | | | |
|---|---|---|---|---|---|---|---|---|---|---|---|---|---|---|---|---|---|---|---|---|
| r_4249 | 'O3-acetyl-L-serine:hydrogen-sulfide 2-amino-2-carboxyethyltransferase; O3-acetyl-L-serine acetate-lyase (adding hydrogen sulfide)' | 8.69E-15 | 4.48E-13 | 4.31E-13 | 4.35E-13 | 4.39E-13 | 4.22E-13 | 4.26E-13 | 1.75E-14 | 1.34E-14 | 4.13E-15 | 1.32E-12 | 50.53713 | 48.51961 | 50.53713 | 3.91E-02 | 2.99E-02 | 9.60E-03 | 149.6725 | 8 |
| r_0950 | 'guanosine phosphorylase' | 4E-14 | -1.61E-12 | -1.62E-12 | -1.63E-12 | 1.65E-12 | 1.66E-12 | 1.67E-12 | 1.04E-14 | 1.87E-14 | 8.26E-15 | 5.01E-12 | 41.24731 | 41.50761 | 41.24731 | 6.47E-03 | 1.16E-02 | 5.10E-03 | 124.0254 | 9 |
| r_0081 | '5-aminolevulinate synthase' | -3.7E-13 | 3.29E-12 | 3.37E-11 | 3.56E-11 | 3.65E-12 | 3.41E-11 | 3.6E-11 | 3.04E-11 | 3.23E-11 | 1.91E-12 | 1.38E-10 | 9.922411 | 92.49235 | 9.922411 | 9.25E+00 | 9.83E+00 | 5.66E-02 | 131.4824 | 10 |
| r_0950 | 'guanosine phosphorylase' | 4E-14 | -1.61E-12 | -1.62E-12 | -1.63E-12 | 1.65E-12 | 1.66E-12 | 1.67E-12 | 1.04E-14 | 1.87E-14 | 8.26E-15 | 5.01E-12 | 41.24731 | 41.50761 | 41.24731 | 6.47E-03 | 1.16E-02 | 5.10E-03 | 124.0254 | 11 |

| | | | | | | | | | | | | | | | | | | | | |
|---|---|---|---|---|---|---|---|---|---|---|---|---|---|---|---|---|---|---|---|---|
| r_1082 | 'uroporphyrinogen methyltransferase' | 1.87E-14 | 7.24E-13 | 7.23E-13 | 7.30E-13 | 7.05E-13 | 7.05E-13 | 7.12E-13 | 7.99E-16 | 6.19E-15 | 6.99E-15 | 2.14E-12 | 37.65119 | 37.60853 | 37.65119 | 1.10E-03 | 8.55E-03 | 9.67E-03 | 112.9302 | 12 |
| r_0210 | 'arginyl-tRNA synthetase' | -1.4E-14 | 4.49E-13 | 4.43E-13 | 4.65E-13 | 4.63E-13 | 4.56E-13 | 4.79E-13 | 6.81E-15 | 1.56E-14 | 2.24E-14 | 1.44E-12 | 34.01312 | 33.51327 | 34.01312 | 1.51E-02 | 3.47E-02 | 5.06E-02 | 101.64 | 13 |
| r_4586 | 'stachyose synthase' | 1.24E-13 | 3.04E-12 | 3.05E-12 | 3.05E-12 | 2.92E-12 | 2.93E-12 | 2.93E-12 | 9.5E-15 | 1.16E-14 | 2.06E-15 | 8.79E-12 | 23.47212 | 23.54858 | 23.47212 | 3.12E-03 | 3.80E-03 | 6.76E-04 | 70.50044 | 14 |
| r_4261 | 'Deaminated glutathione amidase (dGSH amidase) (EC 3.5.1.-) (Nitrilase homolog 1)' | 3.12E-14 | 7.21E-13 | 7.22E-13 | 7.28E-13 | 6.9E-13 | 6.91E-13 | 6.97E-13 | 7.55E-16 | 6.91E-15 | 6.16E-15 | 2.09E-12 | 22.0996 | 22.1238 | 22.0996 | 1.05E-03 | 9.59E-03 | 8.53E-03 | 66.34217 | 15 |
| r_0764 | 'NAD diphosphatase' | 9.92E-15 | 2.26E-13 | 2.27E-13 | 2.38E-13 | 2.16E-13 | 2.17E-13 | 2.29E-13 | 8.9E-16 | 1.28E-14 | 1.2E-14 | 6.87E-13 | 21.73703 | 21.82672 | 21.73703 | 3.94E-03 | 5.70E-02 | 5.28E-02 | 65.41448 | 16 |

| | | | | | | | | | | | | | | | | | | | |
|---|---|---|---|---|---|---|---|---|---|---|---|---|---|---|---|---|---|---|---|
| r_0185 | 'aldehyde dehydrogenase (phenylacetaldehyde, NAD)' | -5.2E-14 | 1.08E-12 | 1.07E-12 | 1.05E-12 | 1.13E-12 | 1.12E-12 | 1.1E-12 | 1.64E-14 | 3.68E-14 | 2.04E-14 | 3.43E-12 | 21.80092 | 21.4858 | 21.80092 | 1.51E-02 | 3.40E-02 | 1.91E-02 | 65.15583 | 17 |
| r_0059 | '3-isopropylmalate 3-methyltransferase' | -2.4E-14 | -5.48E-13 | -4.62E-13 | -5.13E-13 | 5.24E-13 | 4.38E-13 | 4.89E-13 | 8.64E-14 | 3.54E-14 | 5.1E-14 | 1.62E-12 | 21.8765 | 18.2692 | 21.8765 | 1.58E-01 | 6.46E-02 | 1.11E-01 | 62.35501 | 18 |
| r_2589 | 'CL (1-16:0, 2-16:1, 3-16:1, 4-18:1) phospholipase (1-position), mitochondrial membrane' | 1.92E-13 | -2.82E-12 | 5.44E-13 | 9.80E-12 | 3.01E-12 | 3.51E-13 | 9.6E-12 | 3.36E-12 | 1.26E-11 | 9.25E-12 | 3.82E-11 | 15.64267 | 1.825869 | 15.64267 | 1.19E+00 | 4.48E+00 | 1.70E+01 | 55.80552 | 19 |
| r_0332 | 'deoxyribokinase' | 3.01E-14 | -4.74E-13 | -4.69E-13 | -4.69E-13 | 5.04E-13 | 4.99E-13 | 4.99E-13 | 5.56E-15 | 5.59E-15 | 2.5E-17 | 1.51E-12 | 16.73215 | 16.54762 | 16.73215 | 1.17E-02 | 1.18E-02 | 5.33E-05 | 50.03548 | 20 |
